# Supplementary material for: Phosphorylation of Plant Ferredoxin-like Protein Is Required for Intensifying PAMP-Triggered Immunity in Arabidopsis thaliana
Source: Plants (Basel). 2025 Jul 3;14(13):2044. doi: 10.3390/plants14132044 (PMC12251578; doi:10.3390/plants14132044)
Supplement: Supplementary file 1 [file plants-14-02044-s001.zip › plants-3679928-supplementary.pdf]

Supplementary Table S1 Amino acid sequence of recombinant PFLP proteins

Protein	Sequence
PFLP	MASYKVKLITPDGPIEFDCPDDVYILDQAEEAGHDLPYSCRAGSC SSCAGKIAGGAVDQTDGNFLDDDQLEEGWVLTCVAYPQSDVTIE **THKE**AELVG
PFLPT90A	MASYKVKLITPDGPIEFDCPDDVYILDQAEEAGHDLPYSCRAGSC SSCAGKIAGGAVDQTDGNFLDDDQLEEGWVLTCVAYPQSDVTIE **AHKE**AELVG
PFLPT90D	MASYKVKLITPDGPIEFDCPDDVYILDQAEEAGHDLPYSCRAGSC SSCAGKIAGGAVDQTDGNFLDDDQLEEGWVLTCVAYPQSDVTIE **DHKE**AELVG

The underlined position is the amino acid sequence of the CK2P site changed in each mutants from PFLP.

Supplementary Table S2. Primers used in this study

Primer	Sequence	Reference
TUB4-F	5'-agggaaacgaagacagcaag-3'	[1]
TUB4-R	5'-gctcgctaatcctacctttgg-3'	
WRKY22-F	5'-catccgatcaacagacgagtaaat-3'	[2]
WRKY22-R	5'-aaattcgtcggctgaagtcac-3'	
WRKY29-F	5'-tcctatgatcccatccgctg-3'	[2]
WRKY29-R	5'-cgcttggtgcgtactcgtt-3'	
FRK1-F	5'-cggtcagatttcaacagttgtc-3'	[1]
FRK1-R	5'-aatagcaggttggcctgtaatc-3'	
CKA2-XbaI-F	5’- cctctagatgcacctaatc-3’	This study
CKA2-SacI-R	5’- ccgagctctattgagtcctc-3’	This study
35S	5’- aagggatgacgcacaatcccactatccttc-3’	[3]
pBI-IndR	5’- cccagtcacgacgttgtaaa-3’	[3]
50000-F	5'-cctaatagattggggtctcg-3'	This study
50000-R	5'-atcggccgagacatctct-3'	This study
Tub2AF	5'-ctcaagaggttctcagcagta-3'	This study
Tub2AR	5'-tcaccttcttcatccgcagtt-3'	This study

Supplementary Table S3. Plasmids used in this study

Strain or Plasmid	Description	Source
pET16b	Expression vector, Amp^R	Novagen, USA
pET16b-PFLP	Expression vector under control of the T7 promoter containing *pflp* fused to 6x His tag at N-terminal	[4]
pET16b-PFLPT90A	Expression vector under control of the T7 promoter containing *pflpt90a* fused to 6x His tag at N-terminal	This study
pET16b-PFLPT90D	Expression vector under control of the T7 promoter containing *pflpt90d* fused to 6x His tag at N-terminal	This study
pET16b-PEC	Expression vector under control of the T7 promoter containing *pec* fused to 6x His tag at N-terminal	[4]
pET16b-PDC	Expression vector under control of the T7 promoter containing *pdc* fused to 6x His tag at N-terminal	[4]
pGMT-*cka2*	pGTM-T containing from *Arabidopsis thaliana cka2* gene, Amp^R	This study
pBI121- *cka2*	pBI121 containing from *Arabidopsis thaliana cka2* gene, Km^R	This study

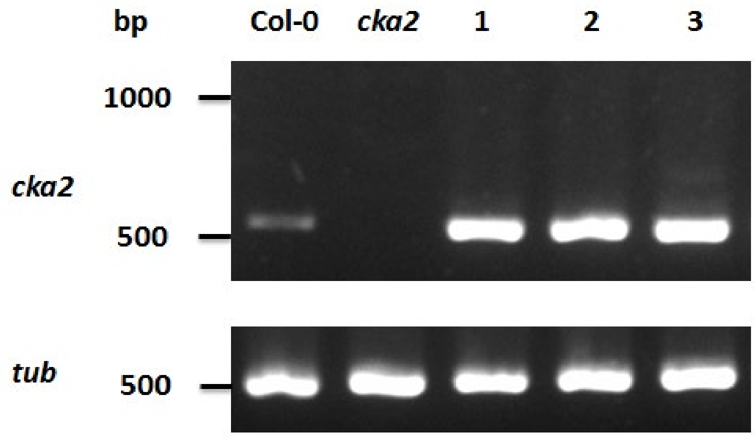

Supplementary Figure S1 Expression of *cka2* transcripts in *Arabidopsis thaliana* CKA2R recovery plants. Leaves of four-week-old plants were collected to analyze expression of TUB2 and *cka2* in Arabidopsis plants. The number of 1, 2 and 3 indicated three CKA2R recovery lines. The PCR product of *TUB2* was 500 bp, and that of *cka2* transcripts was 506 bp. Expression of *TUB2* and *cka2* was confirmed by amplification of cDNA with the specific primers TUB2AF/ TUB2AR, At50000-F/ CKA2-SacI-R. Expression of *TUB2* transcripts was used as positive control.

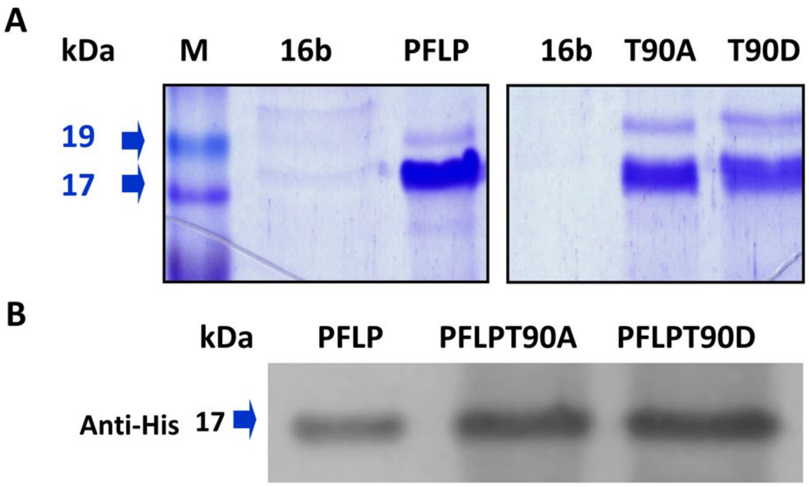

Supplementary Figure S2. Confirmation of purified PFLP recombinant proteins. One microgram of purified recombinant proteins was separated in 15 % resolving gel by SDS-PAGE. Panel A reveals the purified protein solutions separated by SDS-PAGE and then stained with Coomassie blue. Panel B reveals the Western blot analysis of purified derivative recombinant proteins of PFLP. Western blot was performed with anti-His (1:3000) and further stained with DAB. Arrows indicated the protein size at 17 kDa.

References

1. Boudsocq, M.; Willmann, M.R.; McCormack, M.; Lee, H.; Shan, L.; He, P.; Bush, J.; Cheng, S.H.; Sheen, J. Differential innate immune signalling via Ca(2+) sensor protein kinases. *Nature* **2010**, *464*, 418-422, doi:10.1038/nature08794.
2. Göhre, V.; Jones, A.M.E.; Sklenář, J.; Robatzek, S.; Weber, A.P.M. Molecular crosstalk between PAMP-triggered immunity and photosynthesis. *Mol. Plant-Microbe Interact.* **2012**, *25*, 1083-1092.
3. Lin, Y.-H.; Huang, H.-E.; Wu, F.-S.; Ger, M.-J.; Liao, P.-L.; Chen, Y.-R.; Tzeng, K.-C.; Feng, T.-Y. Plant ferredoxin-like protein (PFLP) outside chloroplast in *Arabidopsis* enhances disease resistance against bacterial pathogens. *Plant Sci.* **2010**, *179*, 450-458, doi:10.1016/j.plantsci.2010.07.006.
4. Lin, Y.H.; Huang, H.E.; Chen, Y.R.; Liao, P.L.; Chen, C.L.; Feng, T.Y. C-Terminal Region of Plant Ferredoxin-Like Protein Is Required to Enhance Resistance to Bacterial Disease in. *Phytopathology* **2011**, *101*, 741-749, doi:10.1094/Phyto-08-10-0220.
